# A pilot study to test psychophonetics methodology for self-care and empathy in compassion fatigue, burnout and secondary traumatic stress

**DOI:** 10.4102/phcfm.v5i1.497

**Published:** 2013-09-13

**Authors:** Katherine J. Train, Nadine Butler

**Affiliations:** 1 School of Pharmacy, University of the Western Cape, South Africa

## Abstract

**Background:**

Home-based care is recognised as being a stressful occupation. Practitioners working with patients experiencing high levels of trauma may be susceptible to compassion fatigue, with the sustained need to remain empathic being a contributing factor.

**Objectives:**

The aim of this research was to evaluate psychophonetics methodology for self-care and empathy skills as an intervention for compassion fatigue. Objectives were to measure levels of compassion fatigue pre-intervention, then to apply the intervention and retest levels one month and six months post-intervention.

**Method:**

The research applied a pilot test of a developed intervention as a quasi-experiment. The study sample comprised home-based carers working with HIV-positive patients at a hospice in Grabouw, a settlement in the Western Cape facing socioeconomic challenge.

**Results:**

The result of the pilot study showed a statistically-significant improvement in secondary traumatic stress, a component of compassion fatigue, measured with the ProQOL v5 instrument post-intervention.

**Conclusion:**

The results gave adequate indication for the implementation of a larger study in order to apply and test the intervention. The study highlights a dire need for further research in this field.

## Abstrait

**Une étude pilote visant à tester la méthodologie psychophonétique pour l'auto-prise en charge et l'empathie dans la fatigue de compassion, l’épuisement et le stress traumatique secondaire**

**Contexte:**

La prise en charge à domicile est reconnue comme étant une occupation stressante. Les praticiens travaillant avec des patients connaissant des niveaux élevés de traumatisme peuvent être susceptibles de souffrir de fatigue de compassion, la nécessité continue de faire preuve d'empathie étant un facteur y contribuant.

**Objectifs:**

L'objectif de cette étude consistait à évaluer la méthodologie psychophonétique pour les compétences d'auto-prise en charge et d'empathie, comme intervention contre la fatigue de compassion. Les objectifs consistaient à mesurer les niveaux de fatigue de compassion avant l'intervention, puis à appliquer l'intervention et à tester à nouveau les niveaux un mois et six mois après l'intervention.

**Méthode:**

L’étude a appliqué un test pilote d'une intervention développée comme une quasi-expérimentation. L’échantillon étudié comprenait des soignants à domicile travaillant avec des patients séropositifs dans un hospice à Grabouw, un village du Cap occidental confrontés à des défis socioéconomiques.

**Résultats:**

Le résultat de l’étude pilote a indiqué une amélioration statistiquement significative du stress traumatique secondaire, une composante de la fatigue de compassion, mesurée avec l'instrument ProQOL v5 après l'intervention.

**Conclusion:**

Les résultats ont fourni une indication adéquate pour la mise en œuvre d'une étude de plus grande envergure afin d'appliquer et de tester l'intervention. L’étude met en avant l'absolue nécessité de mener d'autres études dans ce domaine.

## Introduction

### Key focus

The key focus of the research was to study compassion satisfaction and compassion fatigue amongst home-based carers offering palliative care to HIV-positive patients in Grabouw, as well as the testing of an intervention of psychophonetics methodology for self-care and empathy skills in this regard.

### Background

Home-based care, the provision of basic health services to people in their homes, has been recognised as being a stressful occupation.^1, 2, 3^ Various challenges have been identified and may be categorised into physical, emotional, social and financial factors.^3^ Emotional factors have been described as being the recurrence of traumatic images related to the work and thus being distracted whilst at work.^3^

It is well recognised that caregivers working with people who are experiencing, or have experienced, trauma may be susceptible to compassion fatigue.^4, 5, 6, 7^ The sustained need to remain empathic to one's patients has been cited as being a significant factor contributing thereto.^6^ Compassion fatigue may be defined as being a state of tension and arousal experienced by people helping others, by re-experiencing of the traumatic events, numbing and avoidance, or persistent arousal.^8^ It is measured in terms of burnout and secondary traumatic stress.^9^ Offering empathic care to those who have been traumatised may result in an overwhelming, vicarious sharing of the trauma and consequent secondary traumatic stress,^4, 10^ or, as in the case of burnout, a more gradual onset of exhaustion with a resultant need to cut oneself off from those one is caring for, a disengagement from the caring work and a loss of sense of satisfaction and accomplishment with one's work.^7, 10, 11, 12^ It is recognised that caregivers who are able to care for the self are likely to be more resilient to these stressors.^4, 8, 13, 14^

A person may be traumatised both *by being in harm's way* and *by bearing the distress of others who are*. Trauma requires that a severe threatening event has occurred, involving death, injury, or their threat to the person, or to those around them, and includes chronic illness.^15^ Thus, chronic illness such as HIV may be experienced as trauma.

Empathy has been described as being an emotional state or condition that is congruent with another's emotional state or situation.^16, 17^ Empathy may result in an expression of empathic concern or personal distress in the observer.^5, 6, 7^ An unconscious sharing of emotions occurs as emotional resonance,^18^ whilst empathy in a prosocial context requires a moderating cognitive process and an awareness of self in relation to the person being cared for.^18^ The unconscious proclivity to share experiences (including traumatic experiences) between people, if not mediated by self-awareness and cognitive processing, may therefore impact upon the caregiver.

Various studies conducted in Africa have identified empathy as being an associative factor^19, 20^ as well as being the consistent moderating factor^21^ in secondary traumatic stress. These studies were conducted with caregivers responding to patients who had experienced violent crime-related trauma. No distinction was made between empathy as emotional resonance or as a prosocial empathic response in the studies. No studies were available in relation to compassion fatigue in home-based carers.

The psychophonetics parallel counselling process describes a methodology for enabling a prosocial empathic response in two phases: (1) becoming aware of and processing the observer's experience and (2) creating an empathic response as an inner picture in the caregiver of the client's experience.^22^

Psychophonetics is a coaching methodology based on the work of Rudolf Steiner and developed by Yehuda Tagar. In the methodology of psychophonetics, Tagar^23^ identifies three factors, namely awareness of bodily response, internal gesture and visualisation, as being non-verbal aspects of experience that, when brought to consciousness, facilitate an awareness of self. These non-verbal modes of experience are factors that enable an observer to become more aware of the unconscious affective component of emotional resonance and to provide information about this to the observer for further cognitive processing.^23^ The methodology regards the human body as being an instrument of meaning through the mediation of gesture.^23^ When a person observing another mimics the ‘gesture’ or facial, vocal and postural expression of the person being observed, through the process of emotion sharing, the information of this experience becomes available to the observer by the internal sensation of the soma.^24^ Through the mediation of gesture^23^ and the proprioceptive sensory system,^24^ the human body can serve as a map for experience and, through empathy, the experience of the other individual.

For the caregiver to be able to provide a prosocial empathic response and protect themselves from the potentially-deleterious effects of emotion sharing, it is essential that they engage in a parallel internal process of integrating and transforming their experiences.^10^

### Aim and objectives

The aim of the research was to conduct a pilot test of an intervention of psychophonetics self-care and empathy skills and to make a preliminary assessment as to its efficacy in the potential alleviation of compassion fatigue in a study sample of home-based carers employed at a hospice in Grabouw.

The objectives of the research were to measure compassion satisfaction and compassion fatigue in terms of secondary traumatic stress and burnout, using the ProQOL v5 software programme, in the study sample of home-based carers, then to deliver the pilot intervention. Thereafter, the levels were again assessed one month and six months post-intervention, by means of the same instrument.

### Significance of work

This research documents and highlights the potential impact on home-based carers of working with patients and clients who are experiencing, or have experienced, trauma in the form of HIV. In the current socioeconomic climate in South Africa and given the current multiple burden of disease, carers are constantly susceptible to the deleterious effects of patient stress and trauma. It is the researchers’ opinion that this aspect of service delivery in healthcare needs to be given urgent attention as a means to improve healthcare delivery. This investigation aims to highlight the importance of this effect and to stimulate further research in the field.

## Methods

### Research design

The research made use of a participatory action research (PAR) design, applying a preliminary investigation to evaluate the efficacy of a designed intervention.

### Procedure

The methodological elements of psychophonetics for self-care and empathy skills^22^ were applied in a predeveloped format adhering to the theoretical considerations that were deemed to have relevance for prosocial empathic regard.

### Characteristics and training of those delivering the intervention

Facilitators were required to have completed a four-year training Diploma in Psychophonetics and to have at least two years’ experience with group facilitation using the methodology.

### Setting

The two-day intervention (a total of 16 hours) was followed by six 2-hour monthly follow-up supervision sessions, with the mode of delivery being face-to-face, interactive and experiential. Workshop delivery occurred primarily in one large group with exercises where the participants broke into smaller interactive groups of two or three participants, or individual work for reflection or recording of reflections.

### Assumed change process

The assumed change in the participants’ perceptions of key aspects of work and interaction with clients, as related to compassion satisfaction and compassion fatigue (burnout and secondary traumatic stress), was identified and measured using the Professional Quality of Life Scale (ProQOL v5)^9^ instrument. The intervention provided the opportunity for the participants to practise skills that enable a process of self-awareness through self-reflection and to report back within the group.

### Analysis

The study made use of a participating action research design (PAR) with the application of an intervention and a test of repeated measures of the parameters of compassion fatigue on the same subject at three points in time, namely prior to the intervention and then at one month and six months post-intervention.

The ProQOL v5 questionnaires were scored according to the tables in The ProQOL Concise Manual.^9^

### Sampling

The sample comprised 11 women who were employed at the time as home-based carers for a non-profit organisation operating as a hospice in the Grabouw settlement of the Western Cape. All participants were competent in English as a language for training and research. All were within the age range of 18–35 years, had been in the employ of their current employer for less than 5 years and had been in the field of home-based care for less than 5 years. All were undertaking training with the Elgin Learning Foundation in the National Certificate for Ancillary Health Care (equivalent to a matriculation certificate) at the time of the research.

The participants were a pre-existing group, already working as a team. Rabiee^25^ advocates using pre-existing groups, as acquaintances relate to each other's comments and may be more able to challenge each other because of a preformed level of trust, further advancing the advantages of these groups when exploring sensitive issues.

### Instruments

The ProQOL v5 questionnaire provides a measure for compassion satisfaction and compassion fatigue (burnout and secondary traumatic stress). The instrument consists of 30 questions related to aspects of the carer's work perceptions. The questions were divided into three interspersed sections, with 10 questions each related to the carer's perceptions of compassion satisfaction, burnout and secondary traumatic stress. Participants were instructed to rate their experiences of each of the 30 items on a 5-Point Likert scale ranging from 1 (Never) to 5 (Very Often).

For each of the parameters measured, the average score in validation results of other studies was 50, with about 25% of people reporting > 57 and about 25% of people reporting < 43. The results were recorded as total scores for each parameter of compassion satisfaction and compassion fatigue rather than as specific results for each of the items of the questionnaire.

### Reliability and validity of questionnaire

There is good construct validity for the ProQOL v5 questionnaire with nearly half (approximately 50) of the publications on compassion fatigue utilising either the ProQOL v5 questionnaire or one of the earlier versions. The compassion fatigue scale is distinct. The inter-scale correlations show 2% shared variance (*r* = –0.23; co-σ = 5%; *n* = 1187) with secondary traumatic stress and 5% shared variance (*r* = –0.14; co-σ = 2%; *n* = 1187) with burnout.^9^

## Results

A prototype or preliminary intervention was developed into a format that could be applied and evaluated with the home-based carers. A high- and low-risk methodology was then used and cut scores were established based on Stamm's recommendations.^9^

### Pre-intervention

The results relating to the assessment of the susceptibility of the home-based carers to compassion satisfaction and compassion fatigue (burnout and secondary traumatic stress) were recorded before delivery of the intervention (Table 1).

**TABLE 1:** Results of *t*-scores for home-based carers for measures of compassion satisfaction, burnout and secondary traumatic stress using ProQOL v5 (2009) questionnaire before the intervention.

Variable	Compassion satisfaction	Burnout	Secondary traumatic stress
Mean	51.09	56.18	73.72
Median	52	58	76
Range	35–64	45–65	64–77
Low-risk cut	27.27%	Nil	Nil
High-risk cut	18.18%	54.54%	100%

**Compassion satisfaction:** The mean *t*-score was 51.09. The low-risk cut score was scored by 27.27% of the participants and the high-risk cut score by 18.18%. These scores reflect a marginally higher-than-average level of compassion satisfaction than the test group reported by Stamm.^9^

**Burnout:** The mean *t*-score measured for burnout was 56.18. No participants fell within the low-risk cut range and 54.55% of the participants were within the high-risk range. This reflects a high level of risk for burnout amongst the participants in comparison with the test group reported by Stamm.^9^

**Secondary traumatic stress:** The mean *t*-score measured for secondary traumatic stress was 73.72. Five of the analysis group scored the maximum reflected score on the raw to *t*-score conversion table, representing 45% of the group. All participants scored in the high-risk cut range. These results reflect a significantly high risk for secondary traumatic stress amongst the participants tested.

### Post-intervention analysis

Table 2 shows a comparison of *t*-score results from before the intervention, compared with one month after and six months after the intervention. These are also summarised below.

**TABLE 2:** Comparison of *t*-score results for home-based carers for measures of compassion satisfaction, burnout and secondary traumatic stress using ProQOL v5 (2009) questionnaire.

Variable	Compassion satisfaction			Burnout			Secondary traumatic stress		
		
	Bf	+1m	+6m	Bf	+1m	+6m	Bf	+1m	+6m
Mean	51.09	51.90	53.36	56.18	54.30	53.90	73.72	64.30	66.63
Median	52	52	55	58	58	56	76	69	62
Range	35–64	40–64	41–68	45–65	38–59	38–70	64–77	50–77	52–77

Bf ,before the intervention; +1m, one month after the intervention; +6m, six months after the intervention.

**Compassion satisfaction:** The mean *t*-score measured for compassion satisfaction was 51.09 before the delivery of the intervention and had increased to 51.90 by one month post-intervention. Six months after the intervention and following six once-monthly short supervision sessions, the mean *t*-score had increased by 53.36. The lowest score increased from 35 to 41, the median score from 52 to 55 and the highest score from 64 to 68.

**Burnout:** The mean *t*-score measured for burnout was 56.18 before the intervention. One month and six months post-intervention, the mean *t*-scores had decreased by 1.88 points to 54.30 and by 2.28 points to 53.90, respectively. The median score decreased from 58 to 56 and the lowest score from 45 to 38. The highest score decreased from 65 to 59 but increased again to 70.

All of the post-intervention six month scores fell within the range of 38–60, except for one participant. This participant had experienced the death of three close family members during the six-month period of the research. At the time of completion of the final questionnaire, she was experiencing extreme personal trauma and was feeling emotionally overwhelmed.

**Secondary traumatic stress:** The mean *t*-score measured for secondary traumatic stress before the intervention was 73.72. This decreased by 9.42 points (to 64.30) after one month, but only by 7.09 points (to 66.63) after six months. The median score decreased from 76 to 62 and the lowest score decreased from 64 to 50 after one month and to 52 after 6 months. The highest score remained constant at 77.

Two participants who had maintained high scores for secondary traumatic stress throughout the six-month research and intervention period had experienced significant stressful and traumatic experiences in their personal lives during the period.

For compassion satisfaction and for burnout there were no significant differences amongst the periods of pre-intervention, one month post-intervention and six months post-intervention. The *p*-value for the repeated measures analysis of variance was 0.67 for compassion satisfaction and 0.52 for burnout. With regard to secondary traumatic stress, the *p*-value was 0.0066, indicating a significant difference. Pairwise comparisons show that the mean at baseline was different from one month (*p* = 0.0023) and six months (*p* = 0.0193) but the means were not significantly different (*p* = 0.3553).

## Ethical considerations

Ethical approval was granted by the Committee for Research and International Relations, Faculty of Science, University of the Western Cape, reference number ScRIRC2010/01/80. The participants were given free choice regarding whether to participate in the research according to the principles of informed consent. Signed consent was given. The researcher committed to store all records and analyses of the research, either on her person or in a locked cabinet to ensure confidentiality and anonymity of the data. No physical, social or psychological hazards were identified; indeed, there was a potential benefit for the participants associated with their taking part in the intervention.

## Trustworthiness

### Reliability

Internal consistency reliability estimates for the subscales are reported as 0.87 for the compassion satisfaction scale, 0.72 for the burnout scale, and 0.80 for the compassion fatigue/ secondary traumatic stress scale. ^9^

### Validity

There is good construct validity for constructs of compassion fatigue, burnout and secondary traumatic stress with over 200 published papers on the topic and over 100 published research papers on these constructs using the ProQOL or one of its earlier versions as measure. The compassion fatigue scale is distinct with inter-scale correlations showing 2% shared variance with secondary traumatic stress and 5% shared variance with burnout. Whilst there is shared variance between burnout and secondary traumatic stress, they measure two constructs with the shared variance reflecting distress experienced between the scales.^9^

## Discussion

The literature search surrounding the constructs of compassion fatigue, burnout and secondary traumatic stress identified the role of empathy with regard to these conditions. They included the role of the capacity for self-awareness and the ability to regulate the unconscious, affective component of empathy with cognitive processing. These elements, when applied as a parallel internal process by a practitioner or carer, in response to a person being cared for, facilitate the movement from an unconscious, self-protective and defensive response to a prosocial empathic response that is relevant to the person being observed.^22^

Most of the literature on the topic of compassion fatigue links empathy with compassion fatigue, but with conflicting results as to whether it plays an undermining or protective role. Various studies conducted in developed countries, specifically within the context of traumatic episodes such as acts of violence or terrorism,^7, 13^ identify the sustained empathy of a caregiver as contributing to compassion fatigue. Studies conducted in Africa identify empathy as having a protective effect against secondary traumatic stress,^20, 26^ with one study specifically identifying empathy as being the only constant moderator of secondary traumatic stress.^21^ However, these studies do not distinguish between various empathy-related responses such as emotion resonance and prosocial empathy as identified in a recent theoretical paper on the components of empathy.^18^

Psychophonetics methodology for empathy skills was identified by the researcher as applying the components of prosocial empathy in a practical and experiential methodology. The intervention of psychophonetics methodology for self-care and empathy skills focused on encouraging increased levels of self-awareness and body awareness, enabling caregivers to become aware of stressors as they occur, so as to identify the various determining factors, and to become aware of other lifestyle and behavioural options available to them. Making use of a systematic parallel internal process, the methodology trained the participants to identify in themselves the various components of the empathy process.

An increased self-awareness provides the platform for alternative choices to be made. An additional objective of the self-awareness process is to enable participants to have a greater awareness of self and other, as well as the interrelationship between the two. This is to enable the participants to take care of their own needs, allowing them to maintain a state of engagement rather than having to distance themselves from the interaction, in an attempt to protect themselves from further impact, thus enabling a prosocial empathic response, whilst at the same time maintaining protection of themselves.

This study is the first to apply psychophonetics methodology for self-care and empathy skills in relation to compassion fatigue and to measure the results, as no other studies have been recorded in the literature up to now.

The results of the ProQOL v5 questionnaire prior to intervention delivery indicated that the participants were at moderate risk for burnout and high risk for secondary traumatic stress. The groups measured slightly higher scores for job satisfaction than the average of 50 recorded in the research population of more than 1000 used in the development and assessment of the instrument. They measured moderately-higher scores for risk of burnout and significantly-high scores for risk of secondary traumatic stress. The results confirmed quantitatively that the participants were at risk of burnout and secondary traumatic stress.

The range of scores within the group increased in the post-intervention one- and six-month tests for burnout and secondary traumatic stress, compared with the pre-intervention test. The increased range can be attributed to two outliers at the high end of the range. Two participants, experiencing personal distress, maintained high scores for burnout and secondary traumatic stress. Whilst this result may be seen as being a limitation of the instrument and approach, in that it does not record outcomes specific to occupational issues, it alerts one to the possibility of personal factors, specifically personal trauma, contributing toward burnout and secondary traumatic stress in the work context. It further alerts one to the fact that additional mechanisms should be put in place, in an occupational setting, where employees are dealing with extreme client and patient adversity, in order to provide support for them with their own challenges.

## Limitations of the study

Various limitations were identified in relation to the choice and size of the sample. The subjects were employed at the organisation with which the research was planned, meaning that no random sampling occurred. This leads to another limitation of the research, which was the small number of participants in the study group. It is recognised that a small sample size brings into consideration the Hawthorne effect, in that the participants may have altered their behaviours as a result of their awareness of being a part of the study, and also brings into question the participants’ perception of empathy and support from the facilitator during the intervention. The study therefore has limitations regarding translation to a larger population.

A further limitation of this research is the use of the time-series design with only one time-series and one group. The results of the research would have been enhanced if there had been a non-equivalent control group or a multiple time-series design.

## Conclusion

In conclusion, the research determined a significant need amongst the study sample for skills which would allow them to evaluate their risk for compassion fatigue. Whilst the researchers acknowledge the various limitations of the research, the results show enough promise for the implementation of a larger follow-up study with a more rigorous design that would take cognisance of the various limitations identified in this study. The results also highlight a dire need for more awareness of, and research into, this important issue, especially if the people who support those with HIV are to continue their valuable work.
